# Bis[2-(1*H*-1,2,4-triazol-1-yl-κ*N* ^2^)-1,10-phenanthroline-κ^2^ *N*,*N*′]zinc(II) bis­(perchlorate)

**DOI:** 10.1107/S160053680903877X

**Published:** 2009-10-03

**Authors:** Long Miao Xie, Lin Meng, Jing Min Shi

**Affiliations:** a Department of Chemistry, Shandong Normal University, Jinan 250014, People’s Republic of China

## Abstract

In the title complex, [Zn(C_14_H_9_N_5_)_2_](ClO_4_)_2_, 2-(1*H*-1,2,4-triazol-1-yl)-1,10-phenanthroline functions as a tridentate ligand and the Zn^II^ ion assumes a distorted octa­hedral ZnN_6_ coordination geometry. There is a weak π–π stacking inter­action between symmetry-related triazolyl rings with a centroid–centroid distance of 3.802 (4) Å and a perpendicular distance of 3.413 Å between the rings.

## Related literature

For related structures, see: Li (2008 ▶); Liu *et al.* (2008 ▶).
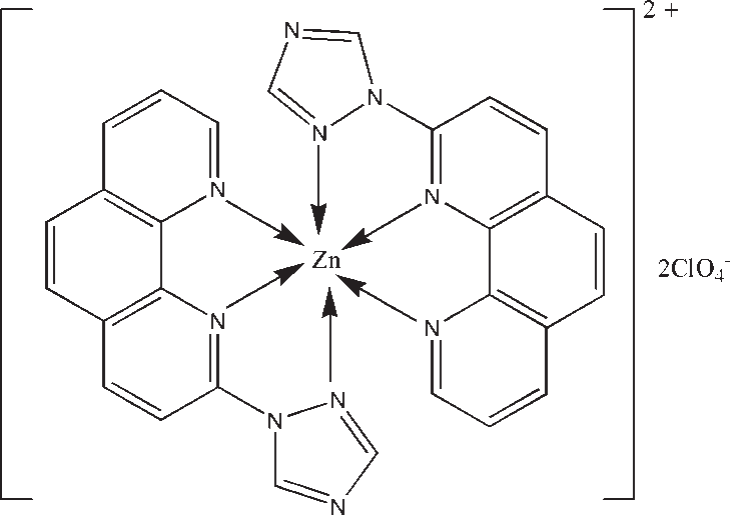


## Experimental

### 

#### Crystal data

[Zn(C_14_H_9_N_5_)_2_](ClO_4_)_2_
*M* *_r_* = 758.79
Monoclinic, 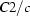

*a* = 16.382 (2) Å
*b* = 26.278 (4) Å
*c* = 15.701 (2) Å
β = 117.399 (2)°
*V* = 6000.8 (14) Å^3^
*Z* = 8
Mo *K*α radiation
μ = 1.07 mm^−1^
*T* = 298 K
0.20 × 0.15 × 0.10 mm

#### Data collection

Bruker SMART APEX CCD diffractometer
Absorption correction: multi-scan (*SADABS*; Sheldrick, 1996 ▶) *T*_min_ = 0.815, *T*_max_ = 0.901
17294 measured reflections
6514 independent reflections
3811 reflections with *I* > 2σ(*I*)
*R*_int_ = 0.068

#### Refinement

*R*[*F* ^2^ > 2σ(*F* ^2^)] = 0.078
*wR*(*F* ^2^) = 0.198
*S* = 1.02
6514 reflections
442 parameters
H-atom parameters constrained
Δρ_max_ = 0.82 e Å^−3^
Δρ_min_ = −0.34 e Å^−3^

### 

Data collection: *SMART* (Bruker, 1997 ▶); cell refinement: *SAINT* (Bruker, 1997 ▶); data reduction: *SAINT*; program(s) used to solve structure: *SHELXS97* (Sheldrick, 2008 ▶); program(s) used to refine structure: *SHELXL97* (Sheldrick, 2008 ▶); molecular graphics: *SHELXTL* (Sheldrick, 2008 ▶); software used to prepare material for publication: *SHELXTL* and local programs.

## Supplementary Material

Crystal structure: contains datablocks I, global. DOI: 10.1107/S160053680903877X/bt5062sup1.cif

Structure factors: contains datablocks I. DOI: 10.1107/S160053680903877X/bt5062Isup2.hkl

Additional supplementary materials:  crystallographic information; 3D view; checkCIF report

## Figures and Tables

**Table:** 

N1—Zn1	2.072 (4)
N2—Zn1	2.235 (4)
N4—Zn1	2.220 (4)
N6—Zn1	2.344 (5)
N9—Zn1	2.079 (4)
N10—Zn1	2.141 (5)

**Table:** 

N1—Zn1—N9	160.34 (17)
N1—Zn1—N10	119.88 (17)
N9—Zn1—N10	77.86 (18)
N1—Zn1—N4	73.44 (15)
N9—Zn1—N4	114.63 (15)
N10—Zn1—N4	98.22 (16)
N1—Zn1—N2	75.83 (15)
N9—Zn1—N2	95.08 (15)
N10—Zn1—N2	95.62 (16)
N4—Zn1—N2	149.23 (15)
N1—Zn1—N6	91.45 (16)
N9—Zn1—N6	71.43 (17)
N10—Zn1—N6	148.63 (17)
N4—Zn1—N6	88.99 (16)
N2—Zn1—N6	93.32 (16)

**Fig. 1.**
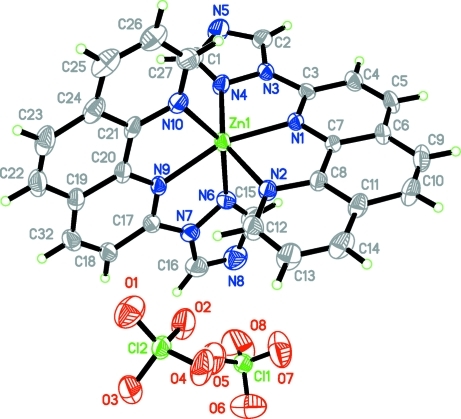
Structure of title compound with the atom-numbering scheme. Displacement ellipsoids are drawn at the 30% probability level.

## supplementary crystallographic information

### Comment

Metal complexes containing derivatives of 1,10-phenanthroline as ligands pay an important role in modern coordination chemistry (Li, 2008 and Liu *et al.* 2008), whereas complexes dealing with 2-(1*H*-1,2,4-triazol-1-yl)-1,10-phenanthroline as ligand have not been published to our knowledge. Interest in this area resulted in us to synthesize a series of this kind of complexes, and herein the crystal structure of the title complex is reported.

Fig. 1 shows the title complex and the data of Table 1 reveal that Zn^II^ ion is located in a distorted octahedral coordination environment. For each 2-(1*H*-1,2,4-triazol-1-yl-κ*N*^2^)-1,10-phenanthroline-k^2^N,*N*' ligand, both 1,2,4-triazolyl ring and 1,10-phenanthroline ring are basically located in a plane within 0.0476 Å (for ligand dealing with atom N1) and 0.0485 Å (for ligand dealing with atom N6) with a maximun deviations of 0.1310 (51) Å and 0.1118 (59) Å for atom C4 and atom C16, respectively, and the dihedral angle between the two planes is 88.07 (5)°, and it means that two planes are nearly perpendicular each other. There is a weak π···π stacking interaction involving symmetry-related 1,2,4-triazolyl rings, with the relevant distances being *Cg*1···*Cg*1^i^ = 3.802 (4) Å and *Cg*1···*Cg*1^i^_perp_ = 3.413 Å [symmetry code: (i) -*x*, *y*, 1/2 - *z*; *Cg*1 is the centroids of C1C2N3-N5 ring].

### Experimental

5 ml Zn(ClO_4_).6H_2_O (0.0473 g, 0.127 mmol) H_2_O solution was added into 10 ml e thanol solution of 2-(1*H*-1,2,4-triazol-1-yl)-1,10-phenanthroline (0.0436 g, 0.176 mmol) and the mixed soluton was stirred for a few minutes. The yellow single crystals were obtained after the filtrate had been allowed to stand at room temperature for two weeks.

### Refinement

All H atoms were placed in calculated positions and refined as riding with C—H = 0.93 Å, *U*_iso_ = 1.2*U*_eq_(C).

### Crystal data

**Table:**

[Zn(C_14_H_9_N_5_)_2_](ClO_4_)_2_	*F*(000) = 3072
*M**_r_* = 758.79	*D*_x_ = 1.680 Mg m^−^^3^
Monoclinic, *C*2/*c*	Mo *K*α radiation, λ = 0.71073 Å
Hall symbol: -C 2yc	Cell parameters from 1862 reflections
*a* = 16.382 (2) Å	θ = 2.6–21.0°
*b* = 26.278 (4) Å	µ = 1.07 mm^−^^1^
*c* = 15.701 (2) Å	*T* = 298 K
β = 117.399 (2)°	Block, yellow
*V* = 6000.8 (14) Å^3^	0.20 × 0.15 × 0.10 mm
*Z* = 8	

### Data collection

**Table:**

Bruker SMART APEX CCD diffractometer	6514 independent reflections
Radiation source: fine-focus sealed tube	3811 reflections with *I* > 2σ(*I*)
graphite	*R*_int_ = 0.068
φ and ω scans	θ_max_ = 27.0°, θ_min_ = 1.6°
Absorption correction: multi-scan (*SADABS*; Sheldrick, 1996)	*h* = −20→11
*T*_min_ = 0.815, *T*_max_ = 0.901	*k* = −33→30
17294 measured reflections	*l* = −19→19

### Refinement

**Table:**

Refinement on *F*^2^	Primary atom site location: structure-invariant direct methods
Least-squares matrix: full	Secondary atom site location: difference Fourier map
*R*[*F*^2^ > 2σ(*F*^2^)] = 0.078	Hydrogen site location: inferred from neighbouring sites
*wR*(*F*^2^) = 0.198	H-atom parameters constrained
*S* = 1.01	*w* = 1/[σ^2^(*F*_o_^2^) + (0.0973*P*)^2^] where *P* = (*F*_o_^2^ + 2*F*_c_^2^)/3
6514 reflections	(Δ/σ)_max_ < 0.001
442 parameters	Δρ_max_ = 0.82 e Å^−^^3^
0 restraints	Δρ_min_ = −0.34 e Å^−^^3^

### Special details

**Table:**

Geometry. All e.s.d.'s (except the e.s.d. in the dihedral angle between two l.s. planes) are estimated using the full covariance matrix. The cell e.s.d.'s are taken into account individually in the estimation of e.s.d.'s in distances, angles and torsion angles; correlations between e.s.d.'s in cell parameters are only used when they are defined by crystal symmetry. An approximate (isotropic) treatment of cell e.s.d.'s is used for estimating e.s.d.'s involving l.s. planes.
Refinement. Refinement of *F*^2^ against ALL reflections. The weighted *R*-factor *wR* and goodness of fit *S* are based on *F*^2^, conventional *R*-factors *R* are based on *F*, with *F* set to zero for negative *F*^2^. The threshold expression of *F*^2^ > σ(*F*^2^) is used only for calculating *R*-factors(gt) *etc*. and is not relevant to the choice of reflections for refinement. *R*-factors based on *F*^2^ are statistically about twice as large as those based on *F*, and *R*- factors based on ALL data will be even larger.

### Fractional atomic coordinates and isotropic or equivalent isotropic displacement parameters (Å^2^)

**Table:**

	*x*	*y*	*z*	*U*_iso_*/*U*_eq_	
C1	0.0631 (4)	0.1459 (2)	0.1825 (4)	0.0624 (17)	
H1	0.0574	0.1811	0.1841	0.075*	
C2	0.0273 (4)	0.0696 (2)	0.1503 (4)	0.0549 (15)	
H2	−0.0047	0.0394	0.1267	0.066*	
C3	0.1886 (3)	0.03571 (18)	0.2340 (3)	0.0368 (12)	
C4	0.1711 (4)	−0.0158 (2)	0.2174 (4)	0.0501 (14)	
H4	0.1114	−0.0285	0.1864	0.060*	
C5	0.2452 (4)	−0.0471 (2)	0.2484 (4)	0.0569 (16)	
H5	0.2363	−0.0820	0.2389	0.068*	
C6	0.3349 (4)	−0.02772 (19)	0.2944 (4)	0.0478 (14)	
C7	0.3436 (3)	0.02411 (18)	0.3104 (3)	0.0374 (12)	
C8	0.4324 (3)	0.04752 (19)	0.3634 (4)	0.0435 (13)	
C9	0.4165 (5)	−0.0573 (2)	0.3268 (5)	0.0703 (19)	
H9	0.4119	−0.0922	0.3156	0.084*	
C10	0.4992 (4)	−0.0360 (2)	0.3727 (5)	0.0675 (18)	
H10	0.5510	−0.0564	0.3911	0.081*	
C11	0.5112 (4)	0.0167 (2)	0.3945 (4)	0.0541 (15)	
C12	0.5163 (4)	0.1190 (2)	0.4324 (5)	0.0604 (16)	
H12	0.5192	0.1536	0.4460	0.072*	
C13	0.5980 (4)	0.0915 (2)	0.4680 (5)	0.0706 (19)	
H13	0.6538	0.1077	0.5052	0.085*	
C14	0.5962 (4)	0.0415 (3)	0.4483 (5)	0.0690 (19)	
H14	0.6509	0.0234	0.4701	0.083*	
C15	0.2538 (4)	0.0882 (2)	0.5021 (5)	0.0575 (16)	
H15	0.2260	0.0569	0.4780	0.069*	
C16	0.3113 (5)	0.1493 (3)	0.5964 (5)	0.0727 (19)	
H16	0.3326	0.1709	0.6492	0.087*	
C17	0.3369 (3)	0.2034 (2)	0.4786 (4)	0.0453 (14)	
C18	0.3637 (4)	0.2490 (2)	0.5325 (4)	0.0580 (17)	
H18	0.3659	0.2517	0.5925	0.070*	
C19	0.3842 (3)	0.2848 (2)	0.4018 (5)	0.0556 (16)	
C20	0.3554 (3)	0.23732 (18)	0.3545 (4)	0.0448 (14)	
C21	0.3478 (3)	0.22907 (19)	0.2619 (4)	0.0465 (14)	
C22	0.4075 (4)	0.3237 (2)	0.3554 (6)	0.075 (2)	
H22	0.4267	0.3551	0.3855	0.090*	
C23	0.4027 (4)	0.3165 (2)	0.2694 (6)	0.076 (2)	
H23	0.4201	0.3428	0.2415	0.091*	
C24	0.3714 (4)	0.2690 (2)	0.2177 (5)	0.0617 (18)	
C25	0.3633 (5)	0.2585 (3)	0.1270 (6)	0.078 (2)	
H25	0.3795	0.2830	0.0949	0.094*	
C26	0.3317 (5)	0.2124 (3)	0.0855 (5)	0.0717 (19)	
H26	0.3265	0.2054	0.0251	0.086*	
C27	0.3073 (4)	0.1755 (2)	0.1339 (4)	0.0592 (16)	
H27	0.2833	0.1447	0.1036	0.071*	
C32	0.3858 (4)	0.2883 (2)	0.4923 (5)	0.067 (2)	
H32	0.4029	0.3190	0.5255	0.081*	
Cl1	0.12715 (11)	0.06126 (6)	0.95141 (13)	0.0646 (5)	
Cl2	0.61091 (10)	0.20923 (5)	0.65565 (12)	0.0611 (4)	
N1	0.2708 (3)	0.05547 (14)	0.2790 (3)	0.0372 (10)	
N2	0.4342 (3)	0.09805 (15)	0.3799 (3)	0.0449 (11)	
N3	0.1183 (3)	0.07318 (15)	0.2035 (3)	0.0423 (10)	
N4	0.1426 (3)	0.12261 (15)	0.2249 (3)	0.0486 (12)	
N5	−0.0102 (3)	0.1146 (2)	0.1362 (4)	0.0689 (15)	
N6	0.2747 (3)	0.12010 (17)	0.4512 (3)	0.0482 (11)	
N7	0.3125 (3)	0.16001 (17)	0.5128 (3)	0.0480 (11)	
N8	0.2756 (4)	0.1038 (2)	0.5924 (4)	0.0813 (17)	
N9	0.3323 (3)	0.19850 (14)	0.3942 (3)	0.0419 (11)	
N10	0.3171 (3)	0.18306 (16)	0.2205 (3)	0.0482 (11)	
O1	0.5926 (4)	0.2410 (2)	0.5782 (4)	0.1038 (18)	
O2	0.5324 (3)	0.18132 (17)	0.6394 (4)	0.0981 (18)	
O3	0.6377 (4)	0.23970 (19)	0.7393 (4)	0.110 (2)	
O4	0.6827 (3)	0.17485 (18)	0.6715 (5)	0.111 (2)	
O5	0.1527 (6)	0.1035 (3)	0.9202 (6)	0.179 (3)	
O6	0.1939 (5)	0.0568 (3)	1.0476 (5)	0.164 (3)	
O7	0.1428 (6)	0.0195 (3)	0.9078 (6)	0.171 (3)	
O8	0.0439 (5)	0.0590 (3)	0.9451 (8)	0.212 (5)	
Zn1	0.29332 (4)	0.13209 (2)	0.31319 (4)	0.0419 (2)	

### Atomic displacement parameters (Å^2^)

**Table:**

	*U*^11^	*U*^22^	*U*^33^	*U*^12^	*U*^13^	*U*^23^
C1	0.042 (4)	0.046 (3)	0.075 (4)	0.008 (3)	0.007 (3)	0.007 (3)
C2	0.036 (3)	0.052 (4)	0.056 (4)	−0.010 (3)	0.004 (3)	0.002 (3)
C3	0.032 (3)	0.038 (3)	0.036 (3)	−0.003 (2)	0.012 (2)	−0.007 (2)
C4	0.051 (4)	0.039 (3)	0.056 (4)	−0.009 (3)	0.021 (3)	−0.007 (3)
C5	0.072 (4)	0.032 (3)	0.064 (4)	−0.008 (3)	0.029 (3)	−0.014 (3)
C6	0.058 (4)	0.031 (3)	0.053 (3)	0.003 (2)	0.025 (3)	−0.002 (2)
C7	0.043 (3)	0.033 (3)	0.033 (3)	0.005 (2)	0.016 (2)	−0.002 (2)
C8	0.044 (3)	0.036 (3)	0.049 (3)	0.006 (2)	0.021 (3)	0.002 (2)
C9	0.079 (5)	0.041 (4)	0.089 (5)	0.016 (3)	0.036 (4)	−0.005 (3)
C10	0.061 (4)	0.049 (4)	0.094 (5)	0.021 (3)	0.037 (4)	0.011 (4)
C11	0.041 (3)	0.050 (3)	0.071 (4)	0.012 (3)	0.026 (3)	0.007 (3)
C12	0.046 (4)	0.046 (3)	0.080 (4)	−0.006 (3)	0.021 (3)	−0.003 (3)
C13	0.034 (3)	0.065 (4)	0.098 (5)	−0.006 (3)	0.017 (3)	0.005 (4)
C14	0.031 (3)	0.075 (5)	0.093 (5)	0.012 (3)	0.023 (3)	0.016 (4)
C15	0.045 (4)	0.058 (4)	0.063 (4)	0.009 (3)	0.020 (3)	0.013 (3)
C16	0.086 (5)	0.078 (5)	0.051 (4)	0.014 (4)	0.029 (4)	−0.001 (4)
C17	0.030 (3)	0.041 (3)	0.051 (4)	0.009 (2)	0.007 (2)	−0.006 (3)
C18	0.047 (4)	0.043 (3)	0.061 (4)	0.011 (3)	0.004 (3)	−0.009 (3)
C19	0.029 (3)	0.034 (3)	0.079 (5)	0.000 (2)	0.003 (3)	0.002 (3)
C20	0.026 (3)	0.033 (3)	0.064 (4)	0.000 (2)	0.011 (2)	0.003 (3)
C21	0.031 (3)	0.032 (3)	0.065 (4)	0.004 (2)	0.013 (3)	0.013 (3)
C22	0.055 (4)	0.038 (4)	0.103 (6)	−0.005 (3)	0.012 (4)	−0.001 (4)
C23	0.046 (4)	0.044 (4)	0.117 (7)	−0.004 (3)	0.021 (4)	0.019 (4)
C24	0.039 (3)	0.054 (4)	0.083 (5)	0.004 (3)	0.020 (3)	0.024 (3)
C25	0.063 (5)	0.090 (6)	0.086 (6)	0.015 (4)	0.037 (4)	0.038 (5)
C26	0.072 (5)	0.078 (5)	0.070 (5)	0.011 (4)	0.037 (4)	0.025 (4)
C27	0.059 (4)	0.056 (4)	0.054 (4)	−0.002 (3)	0.019 (3)	0.004 (3)
C32	0.042 (4)	0.048 (4)	0.078 (5)	0.007 (3)	−0.002 (3)	−0.020 (3)
Cl1	0.0555 (10)	0.0612 (10)	0.0722 (11)	−0.0084 (7)	0.0251 (8)	−0.0016 (8)
Cl2	0.0475 (9)	0.0434 (8)	0.0758 (11)	0.0065 (6)	0.0140 (7)	0.0022 (8)
N1	0.035 (2)	0.031 (2)	0.040 (2)	−0.0009 (17)	0.0125 (18)	−0.0021 (18)
N2	0.035 (2)	0.036 (2)	0.055 (3)	−0.0022 (18)	0.013 (2)	−0.001 (2)
N3	0.039 (3)	0.033 (2)	0.044 (3)	−0.0050 (18)	0.010 (2)	−0.0052 (19)
N4	0.037 (3)	0.035 (2)	0.058 (3)	−0.0018 (18)	0.008 (2)	0.005 (2)
N5	0.035 (3)	0.059 (3)	0.086 (4)	−0.002 (2)	0.005 (3)	0.008 (3)
N6	0.044 (3)	0.049 (3)	0.046 (3)	−0.002 (2)	0.015 (2)	−0.003 (2)
N7	0.041 (3)	0.051 (3)	0.046 (3)	0.008 (2)	0.015 (2)	0.000 (2)
N8	0.097 (5)	0.081 (5)	0.074 (4)	0.010 (3)	0.046 (4)	0.014 (4)
N9	0.031 (2)	0.030 (2)	0.053 (3)	0.0021 (17)	0.009 (2)	−0.003 (2)
N10	0.044 (3)	0.045 (3)	0.051 (3)	0.002 (2)	0.018 (2)	0.007 (2)
O1	0.129 (5)	0.108 (4)	0.094 (4)	0.031 (3)	0.069 (4)	0.030 (3)
O2	0.046 (3)	0.064 (3)	0.155 (5)	−0.001 (2)	0.022 (3)	0.009 (3)
O3	0.164 (6)	0.064 (3)	0.081 (4)	0.001 (3)	0.037 (4)	−0.009 (3)
O4	0.050 (3)	0.066 (3)	0.194 (6)	0.021 (2)	0.036 (3)	0.013 (3)
O5	0.220 (9)	0.107 (6)	0.183 (7)	−0.039 (5)	0.071 (6)	0.051 (5)
O6	0.137 (6)	0.225 (9)	0.109 (5)	−0.035 (6)	0.038 (5)	0.010 (5)
O7	0.259 (10)	0.101 (5)	0.202 (8)	0.009 (6)	0.147 (7)	−0.021 (5)
O8	0.074 (5)	0.219 (9)	0.332 (12)	−0.024 (5)	0.084 (6)	−0.080 (8)
Zn1	0.0370 (4)	0.0292 (3)	0.0523 (4)	−0.0038 (2)	0.0143 (3)	−0.0033 (3)

### Geometric parameters (Å, °)

**Table:**

C1—N4	1.310 (7)	C17—C18	1.417 (7)
C1—N5	1.359 (7)	C18—C32	1.342 (9)
C1—H1	0.9300	C18—H18	0.9300
C2—N5	1.302 (7)	C19—C22	1.405 (9)
C2—N3	1.335 (6)	C19—C32	1.413 (9)
C2—H2	0.9300	C19—C20	1.418 (7)
C3—N1	1.306 (6)	C20—N9	1.337 (6)
C3—C4	1.384 (7)	C20—C21	1.416 (8)
C3—N3	1.420 (6)	C21—N10	1.355 (6)
C4—C5	1.357 (7)	C21—C24	1.408 (7)
C4—H4	0.9300	C22—C23	1.330 (10)
C5—C6	1.401 (7)	C22—H22	0.9300
C5—H5	0.9300	C23—C24	1.448 (9)
C6—C7	1.381 (7)	C23—H23	0.9300
C6—C9	1.423 (8)	C24—C25	1.395 (10)
C7—N1	1.343 (6)	C25—C26	1.360 (9)
C7—C8	1.440 (7)	C25—H25	0.9300
C8—N2	1.351 (6)	C26—C27	1.399 (8)
C8—C11	1.407 (7)	C26—H26	0.9300
C9—C10	1.331 (8)	C27—N10	1.309 (7)
C9—H9	0.9300	C27—H27	0.9300
C10—C11	1.417 (8)	C32—H32	0.9300
C10—H10	0.9300	Cl1—O8	1.323 (7)
C11—C14	1.413 (8)	Cl1—O5	1.356 (6)
C12—N2	1.331 (7)	Cl1—O7	1.379 (7)
C12—C13	1.391 (8)	Cl1—O6	1.403 (7)
C12—H12	0.9300	Cl2—O1	1.390 (5)
C13—C14	1.347 (8)	Cl2—O2	1.398 (5)
C13—H13	0.9300	Cl2—O4	1.411 (4)
C14—H14	0.9300	Cl2—O3	1.424 (5)
C15—N6	1.308 (7)	N1—Zn1	2.072 (4)
C15—N8	1.357 (8)	N2—Zn1	2.235 (4)
C15—H15	0.9300	N3—N4	1.355 (5)
C16—N8	1.321 (9)	N4—Zn1	2.220 (4)
C16—N7	1.350 (7)	N6—N7	1.367 (6)
C16—H16	0.9300	N6—Zn1	2.344 (5)
C17—N9	1.299 (7)	N9—Zn1	2.079 (4)
C17—N7	1.395 (7)	N10—Zn1	2.141 (5)
			
N4—C1—N5	114.6 (5)	C25—C24—C21	115.9 (6)
N4—C1—H1	122.7	C25—C24—C23	125.8 (7)
N5—C1—H1	122.7	C21—C24—C23	118.2 (7)
N5—C2—N3	110.3 (5)	C26—C25—C24	120.1 (7)
N5—C2—H2	124.8	C26—C25—H25	119.9
N3—C2—H2	124.8	C24—C25—H25	119.9
N1—C3—C4	124.3 (5)	C25—C26—C27	119.8 (7)
N1—C3—N3	112.4 (4)	C25—C26—H26	120.1
C4—C3—N3	123.4 (4)	C27—C26—H26	120.1
C5—C4—C3	116.8 (5)	N10—C27—C26	122.0 (6)
C5—C4—H4	121.6	N10—C27—H27	119.0
C3—C4—H4	121.6	C26—C27—H27	119.0
C4—C5—C6	121.2 (5)	C18—C32—C19	122.6 (6)
C4—C5—H5	119.4	C18—C32—H32	118.7
C6—C5—H5	119.4	C19—C32—H32	118.7
C7—C6—C5	116.6 (5)	O8—Cl1—O5	119.2 (6)
C7—C6—C9	118.2 (5)	O8—Cl1—O7	110.4 (5)
C5—C6—C9	125.2 (5)	O5—Cl1—O7	108.3 (5)
N1—C7—C6	122.6 (5)	O8—Cl1—O6	110.0 (6)
N1—C7—C8	116.2 (4)	O5—Cl1—O6	103.9 (5)
C6—C7—C8	121.3 (5)	O7—Cl1—O6	103.7 (5)
N2—C8—C11	124.3 (5)	O1—Cl2—O2	110.2 (3)
N2—C8—C7	117.2 (4)	O1—Cl2—O4	111.5 (4)
C11—C8—C7	118.4 (5)	O2—Cl2—O4	108.6 (3)
C10—C9—C6	121.2 (6)	O1—Cl2—O3	108.6 (3)
C10—C9—H9	119.4	O2—Cl2—O3	109.3 (4)
C6—C9—H9	119.4	O4—Cl2—O3	108.6 (4)
C9—C10—C11	122.4 (6)	C3—N1—C7	118.4 (4)
C9—C10—H10	118.8	C3—N1—Zn1	122.9 (3)
C11—C10—H10	118.8	C7—N1—Zn1	118.6 (3)
C8—C11—C14	115.8 (5)	C12—N2—C8	116.8 (5)
C8—C11—C10	118.4 (5)	C12—N2—Zn1	131.0 (4)
C14—C11—C10	125.8 (5)	C8—N2—Zn1	112.2 (3)
N2—C12—C13	123.1 (6)	C2—N3—N4	109.8 (4)
N2—C12—H12	118.4	C2—N3—C3	131.4 (4)
C13—C12—H12	118.4	N4—N3—C3	118.7 (4)
C14—C13—C12	119.9 (6)	C1—N4—N3	102.2 (4)
C14—C13—H13	120.1	C1—N4—Zn1	145.6 (4)
C12—C13—H13	120.1	N3—N4—Zn1	112.2 (3)
C13—C14—C11	120.0 (5)	C2—N5—C1	103.1 (5)
C13—C14—H14	120.0	C15—N6—N7	102.7 (5)
C11—C14—H14	120.0	C15—N6—Zn1	146.4 (4)
N6—C15—N8	115.3 (6)	N7—N6—Zn1	110.1 (3)
N6—C15—H15	122.3	C16—N7—N6	109.0 (5)
N8—C15—H15	122.3	C16—N7—C17	132.8 (5)
N8—C16—N7	110.2 (6)	N6—N7—C17	118.0 (4)
N8—C16—H16	124.9	C16—N8—C15	102.8 (6)
N7—C16—H16	124.9	C17—N9—C20	120.3 (5)
N9—C17—N7	114.9 (5)	C17—N9—Zn1	124.3 (4)
N9—C17—C18	123.3 (6)	C20—N9—Zn1	115.4 (4)
N7—C17—C18	121.8 (6)	C27—N10—C21	118.4 (5)
C32—C18—C17	116.5 (6)	C27—N10—Zn1	129.6 (4)
C32—C18—H18	121.7	C21—N10—Zn1	112.0 (4)
C17—C18—H18	121.7	N1—Zn1—N9	160.34 (17)
C22—C19—C32	126.3 (6)	N1—Zn1—N10	119.88 (17)
C22—C19—C20	118.2 (6)	N9—Zn1—N10	77.86 (18)
C32—C19—C20	115.6 (6)	N1—Zn1—N4	73.44 (15)
N9—C20—C21	116.8 (5)	N9—Zn1—N4	114.63 (15)
N9—C20—C19	121.6 (6)	N10—Zn1—N4	98.22 (16)
C21—C20—C19	121.6 (5)	N1—Zn1—N2	75.83 (15)
N10—C21—C24	123.6 (6)	N9—Zn1—N2	95.08 (15)
N10—C21—C20	117.9 (5)	N10—Zn1—N2	95.62 (16)
C24—C21—C20	118.5 (5)	N4—Zn1—N2	149.23 (15)
C23—C22—C19	121.3 (6)	N1—Zn1—N6	91.45 (16)
C23—C22—H22	119.3	N9—Zn1—N6	71.43 (17)
C19—C22—H22	119.3	N10—Zn1—N6	148.63 (17)
C22—C23—C24	122.1 (7)	N4—Zn1—N6	88.99 (16)
C22—C23—H23	118.9	N2—Zn1—N6	93.32 (16)
C24—C23—H23	118.9		
			
N1—C3—C4—C5	−1.6 (8)	C15—N6—N7—C16	−0.2 (6)
N3—C3—C4—C5	178.2 (5)	Zn1—N6—N7—C16	−172.4 (4)
C3—C4—C5—C6	−0.4 (8)	C15—N6—N7—C17	−175.4 (4)
C4—C5—C6—C7	2.9 (8)	Zn1—N6—N7—C17	12.4 (5)
C4—C5—C6—C9	−178.1 (6)	N9—C17—N7—C16	177.3 (5)
C5—C6—C7—N1	−3.8 (8)	C18—C17—N7—C16	−3.2 (9)
C9—C6—C7—N1	177.1 (5)	N9—C17—N7—N6	−8.8 (6)
C5—C6—C7—C8	175.4 (5)	C18—C17—N7—N6	170.6 (4)
C9—C6—C7—C8	−3.7 (8)	N7—C16—N8—C15	−1.1 (7)
N1—C7—C8—N2	2.7 (7)	N6—C15—N8—C16	1.0 (7)
C6—C7—C8—N2	−176.6 (5)	N7—C17—N9—C20	−179.1 (4)
N1—C7—C8—C11	−177.4 (5)	C18—C17—N9—C20	1.5 (7)
C6—C7—C8—C11	3.3 (8)	N7—C17—N9—Zn1	−0.6 (6)
C7—C6—C9—C10	1.1 (9)	C18—C17—N9—Zn1	−180.0 (4)
C5—C6—C9—C10	−177.9 (6)	C21—C20—N9—C17	−179.5 (4)
C6—C9—C10—C11	1.8 (11)	C19—C20—N9—C17	−0.8 (7)
N2—C8—C11—C14	1.5 (9)	C21—C20—N9—Zn1	1.9 (6)
C7—C8—C11—C14	−178.3 (5)	C19—C20—N9—Zn1	−179.5 (4)
N2—C8—C11—C10	179.5 (6)	C26—C27—N10—C21	3.2 (8)
C7—C8—C11—C10	−0.3 (8)	C26—C27—N10—Zn1	−175.8 (4)
C9—C10—C11—C8	−2.2 (10)	C24—C21—N10—C27	−1.2 (7)
C9—C10—C11—C14	175.6 (6)	C20—C21—N10—C27	178.1 (5)
N2—C12—C13—C14	1.0 (11)	C24—C21—N10—Zn1	178.0 (4)
C12—C13—C14—C11	−2.2 (11)	C20—C21—N10—Zn1	−2.7 (5)
C8—C11—C14—C13	1.0 (9)	C3—N1—Zn1—N9	111.5 (5)
C10—C11—C14—C13	−176.8 (7)	C7—N1—Zn1—N9	−63.6 (6)
N9—C17—C18—C32	−0.6 (7)	C3—N1—Zn1—N10	−95.9 (4)
N7—C17—C18—C32	180.0 (5)	C7—N1—Zn1—N10	89.0 (4)
C22—C19—C20—N9	179.8 (5)	C3—N1—Zn1—N4	−5.9 (4)
C32—C19—C20—N9	−0.6 (7)	C7—N1—Zn1—N4	179.0 (4)
C22—C19—C20—C21	−1.6 (7)	C3—N1—Zn1—N2	175.7 (4)
C32—C19—C20—C21	178.0 (5)	C7—N1—Zn1—N2	0.6 (4)
N9—C20—C21—N10	0.7 (7)	C3—N1—Zn1—N6	82.7 (4)
C19—C20—C21—N10	−178.0 (4)	C7—N1—Zn1—N6	−92.4 (4)
N9—C20—C21—C24	180.0 (5)	C17—N9—Zn1—N1	−25.2 (7)
C19—C20—C21—C24	1.3 (7)	C20—N9—Zn1—N1	153.4 (4)
C32—C19—C22—C23	−179.4 (6)	C17—N9—Zn1—N10	178.9 (4)
C20—C19—C22—C23	0.2 (9)	C20—N9—Zn1—N10	−2.5 (3)
C19—C22—C23—C24	1.4 (10)	C17—N9—Zn1—N4	85.3 (4)
N10—C21—C24—C25	−1.3 (8)	C20—N9—Zn1—N4	−96.1 (4)
C20—C21—C24—C25	179.4 (5)	C17—N9—Zn1—N2	−86.4 (4)
N10—C21—C24—C23	179.6 (5)	C20—N9—Zn1—N2	92.2 (3)
C20—C21—C24—C23	0.3 (8)	C17—N9—Zn1—N6	5.4 (4)
C22—C23—C24—C25	179.3 (6)	C20—N9—Zn1—N6	−176.0 (4)
C22—C23—C24—C21	−1.7 (9)	C27—N10—Zn1—N1	10.9 (5)
C21—C24—C25—C26	1.8 (9)	C21—N10—Zn1—N1	−168.1 (3)
C23—C24—C25—C26	−179.2 (6)	C27—N10—Zn1—N9	−178.2 (5)
C24—C25—C26—C27	0.1 (10)	C21—N10—Zn1—N9	2.7 (3)
C25—C26—C27—N10	−2.7 (10)	C27—N10—Zn1—N4	−64.6 (5)
C17—C18—C32—C19	−0.9 (8)	C21—N10—Zn1—N4	116.3 (3)
C22—C19—C32—C18	−179.0 (6)	C27—N10—Zn1—N2	87.8 (5)
C20—C19—C32—C18	1.4 (8)	C21—N10—Zn1—N2	−91.3 (3)
C4—C3—N1—C7	0.9 (8)	C27—N10—Zn1—N6	−166.3 (4)
N3—C3—N1—C7	−179.0 (4)	C21—N10—Zn1—N6	14.6 (5)
C4—C3—N1—Zn1	−174.2 (4)	C1—N4—Zn1—N1	−175.7 (8)
N3—C3—N1—Zn1	5.9 (6)	N3—N4—Zn1—N1	4.4 (3)
C6—C7—N1—C3	2.0 (7)	C1—N4—Zn1—N9	23.5 (8)
C8—C7—N1—C3	−177.3 (4)	N3—N4—Zn1—N9	−156.4 (3)
C6—C7—N1—Zn1	177.3 (4)	C1—N4—Zn1—N10	−56.9 (8)
C8—C7—N1—Zn1	−1.9 (6)	N3—N4—Zn1—N10	123.2 (4)
C13—C12—N2—C8	1.4 (9)	C1—N4—Zn1—N2	−172.7 (7)
C13—C12—N2—Zn1	−179.5 (5)	N3—N4—Zn1—N2	7.4 (6)
C11—C8—N2—C12	−2.6 (8)	C1—N4—Zn1—N6	92.5 (8)
C7—C8—N2—C12	177.2 (5)	N3—N4—Zn1—N6	−87.4 (4)
C11—C8—N2—Zn1	178.1 (4)	C12—N2—Zn1—N1	−178.3 (6)
C7—C8—N2—Zn1	−2.1 (6)	C8—N2—Zn1—N1	0.8 (4)
N5—C2—N3—N4	−0.7 (7)	C12—N2—Zn1—N9	−16.0 (5)
N5—C2—N3—C3	−176.8 (5)	C8—N2—Zn1—N9	163.1 (4)
N1—C3—N3—C2	174.5 (5)	C12—N2—Zn1—N10	62.2 (5)
C4—C3—N3—C2	−5.3 (9)	C8—N2—Zn1—N10	−118.6 (4)
N1—C3—N3—N4	−1.2 (6)	C12—N2—Zn1—N4	178.7 (5)
C4—C3—N3—N4	178.9 (5)	C8—N2—Zn1—N4	−2.1 (6)
N5—C1—N4—N3	0.4 (7)	C12—N2—Zn1—N6	−87.7 (5)
N5—C1—N4—Zn1	−179.5 (5)	C8—N2—Zn1—N6	91.5 (4)
C2—N3—N4—C1	0.2 (6)	C15—N6—Zn1—N1	−5.0 (7)
C3—N3—N4—C1	176.9 (5)	N7—N6—Zn1—N1	161.1 (3)
C2—N3—N4—Zn1	−179.9 (4)	C15—N6—Zn1—N9	−175.1 (7)
C3—N3—N4—Zn1	−3.2 (6)	N7—N6—Zn1—N9	−9.0 (3)
N3—C2—N5—C1	0.9 (7)	C15—N6—Zn1—N10	172.6 (6)
N4—C1—N5—C2	−0.8 (8)	N7—N6—Zn1—N10	−21.2 (5)
N8—C15—N6—N7	−0.5 (6)	C15—N6—Zn1—N4	68.4 (7)
N8—C15—N6—Zn1	166.1 (5)	N7—N6—Zn1—N4	−125.5 (3)
N8—C16—N7—N6	0.9 (7)	C15—N6—Zn1—N2	−80.9 (7)
N8—C16—N7—C17	175.1 (5)	N7—N6—Zn1—N2	85.2 (3)
